# Going deeper into the toxicokinetics of synthetic cannabinoids: in vitro contribution of human carboxylesterases

**DOI:** 10.1007/s00204-022-03332-z

**Published:** 2022-07-05

**Authors:** Lea Wagmann, Rebecca G. Stiller, Svenja Fischmann, Folker Westphal, Markus R. Meyer

**Affiliations:** 1 Department of Experimental and Clinical Toxicology, Institute of Experimental and Clinical Pharmacology and Toxicology, Center for Molecular Signaling (PZMS), Saarland University, Homburg, Germany; 2 State Bureau of Criminal Investigation Schleswig-Holstein, Kiel, Germany

**Keywords:** Drugs of abuse, NPS, Enzyme kinetics, Esterases, Metabolism, LC–ITMS

## Abstract

Synthetic cannabinoids (SC) are new psychoactive substances known to cause intoxications and fatalities. One reason may be the limited data available concerning the toxicokinetics of SC, but toxicity mechanisms are insufficiently understood so far. Human carboxylesterases (hCES) are widely known to play a crucial role in the catalytic hydrolysis of drugs (of abuse). The aim of this study was to investigate the in vitro contribution of hCES to the metabolism of the 13 SC 3,5-AB-5F-FUPPYCA, AB-5F-P7AICA, A-CHMINACA, DMBA-CHMINACA, MBA-CHMINACA, MDMB-4F-BINACA, MDMB-4en-PINACA, MDMB-FUBICA, MDMB-5F-PICA, MMB-CHMICA, MMB-4en-PICA, MMB-FUBINACA, and MPhP-5F-PICA. The SC were incubated with recombinant hCES1b, hCES1c, or hCES2 and analyzed by liquid chromatography–ion trap mass spectrometry to assess amide or ester hydrolysis in an initial activity screening. Enzyme kinetic studies were performed if sufficient hydrolysis was observed. No hydrolysis of the amide linker was observed using those experimental conditions. Except for MDMB-5F-PICA, ester hydrolysis was always detected if an ester group was present in the head group. In general, SC with a terminal ester bearing a small alcohol part and a larger acyl part showed higher affinity to hCES1 isozymes. Due to the low hydrolysis rates, enzyme kinetics could not be modeled for the SC with a *tert*-leucine-derived moiety, but hydrolysis reactions of MPhP-5F-PICA and of those containing a valine-derived moiety followed classic Michaelis–Menten kinetics. In conclusion, drug–drug/drug–food interactions or hCES polymorphisms may prolong the half-life of SC and the current results help to estimate the risk of toxicity in the future after combining them with activity and clinical data.

**Supplementary Information:**

The online version contains supplementary material available at 10.1007/s00204-022-03332-z.

## Introduction

The steadily increasing number of new psychoactive substances (NPS) pushed the diversity and complexity of the drugs of abuse market during the last decade. More than 500 different NPS are reported to the United Nations Office on Drugs and Crime each year and the synthetic cannabinoids (SC) are one of the largest subgroups (UNODC 2021). SC interact with human cannabinoid type 1 (CB1) and/or type 2 (CB2) receptors and are expected to induce similar effects as phytocannabinoids found in *Cannabis sativa* such as tetrahydrocannabinol (Banister and Connor 2018). Even though the psychotropic effects of SC are comparable to those of cannabis, severe and fatal poisonings are more common after consumption of SC (Tait et al. 2016). Symptoms include, but are not limited to, cardiovascular toxicity, rapid loss of consciousness, coma, respiratory depression, and seizures (EMCDDA 2018). Not only the acute, but also the chronic effects of SC exposure cause concern among the scientific community as the endogenous cannabinoid system plays an important role in human health and behavior (Diao and Huestis 2019). Unfortunately, toxicity mechanisms are insufficiently understood so far. On the one hand, many SC are full agonists at the cannabinoid receptors with a higher potency compared to the partial agonist tetrahydrocannabinol (EMCDDA 2020; Ford et al. 2017). On the other hand, only limited data concerning the toxicokinetics of SC are available. However, the formation of active metabolites during SC biotransformation is expected to play a crucial role in toxicity. Cannaert et al. tested SC and several metabolites in a CB1 and CB2 receptor activation assay and reported, that the phase I metabolites retain activity at the cannabinoid receptors (Cannaert et al. 2017). For example, the carboxylic acid metabolite of the SC AB-CHMINACA displayed a significantly stronger level of CB1 receptor activation than the full agonist JWH-018 (Cannaert et al. 2017).

**Fig. 1:**
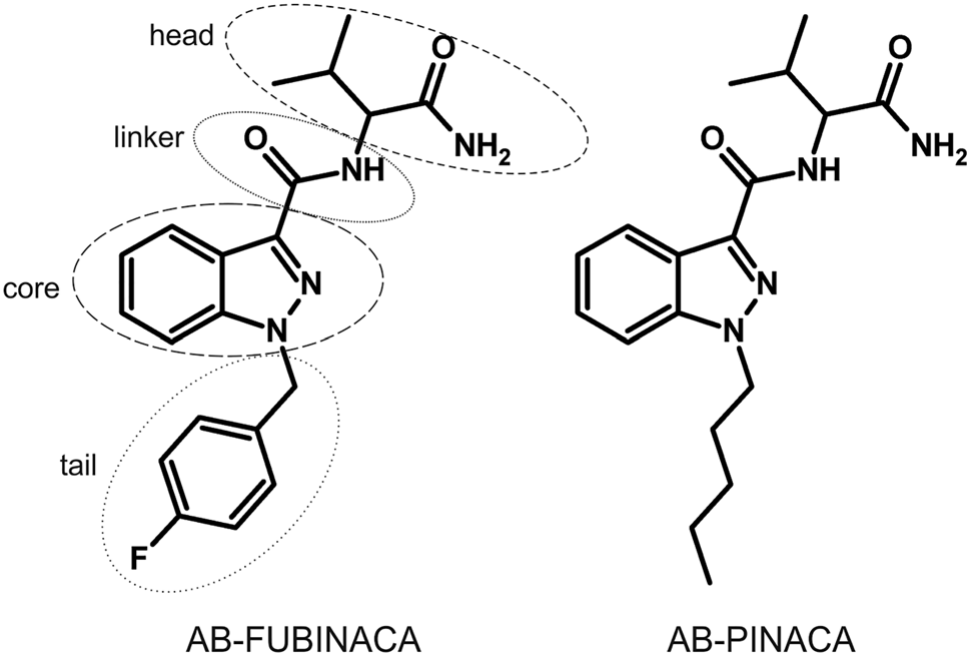
Chemical structures of AB-FUBINACA and AB-PINACA and indication of the four subunits in the structure of synthetic cannabinoids

In 2015, Thomsen et al. identified human carboxylesterases (hCES) to be involved in the formation of the carboxylic acid metabolites of the SC AB-FUBINACA and AB-PINACA (structures see Fig. 1) (Thomsen et al. 2015). Especially, the hCES of the subfamilies 1 and 2 play a crucial role in the catalytic hydrolysis of exogenous substances containing ester, amide, carbamate, or thioester moieties (Di 2019). While hCES2 is primarily expressed in the gastrointestinal tract and kidneys, hCES1 is predominantly located in liver and lungs (Di 2019; Imai et al. 2006). In the liver, the main drug-metabolizing organ in humans, expression levels of hCES1 are even higher than those of cytochrome P450 and UDP-glucuronosyltransferase isoforms (He et al. 2019; Qian et al. 2020). Two isoforms of hCES1, by name hCES1b and hCES1c, were identified. These isoforms differ in some point mutations, and, therefore, in their substrate specificity with hCES1b being the isoform which is predominant in the human liver (Di 2019; Wang et al. 2011). Investigations on the phase I metabolism of drugs and drugs of abuse demonstrated, that hCES may be essential for both bioactivation and inactivation processes. While for example, the detoxification of cocaine is catalyzed by hCES, prodrugs such as imidapril need to be activated by hCES-driven hydrolysis (Imai et al. 2006; Merali et al. 2014).

**Fig. 2:**
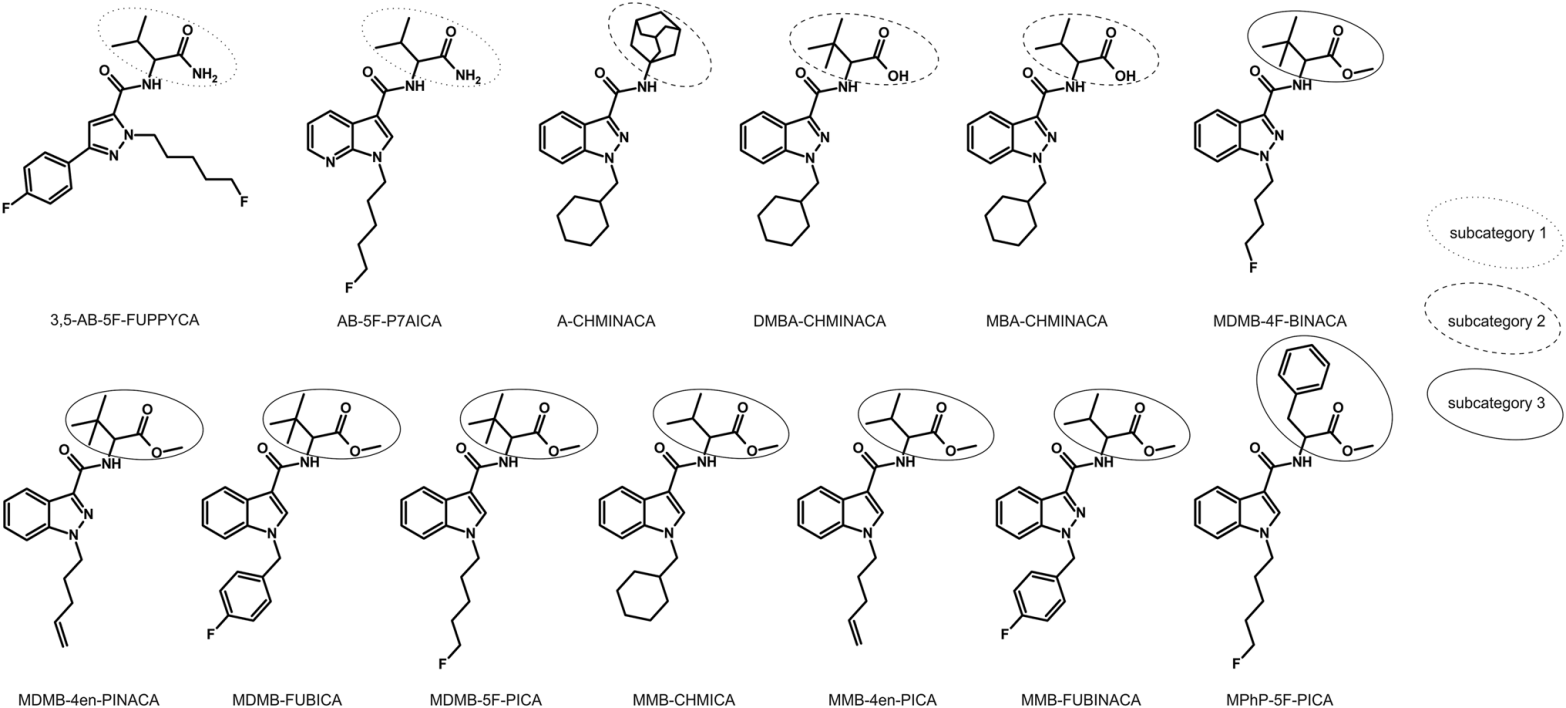
Chemical structures of the 13 tested synthetic cannabinoids including subcategory classification

While numerous publications about the involvement of hCES in the metabolism of drugs are available, only few studies have been conducted demonstrating that hCES are also involved in the bioactivation or -inactivation of drugs of abuse such as cocaine, opiates, and alkaloids (Hatfield et al. 2010; Meyer et al. 2015; Yao et al. 2018). Even less reports are available concerning their role in the metabolic fate of NPS (Richter et al. 2021; Thomsen et al. 2015; Wagmann et al. 2020). All NPS-related publications described the involvement of hCES in the metabolism of single SC. The aim of this study was to enlarge the knowledge concerning the in vitro contribution of hCES to the metabolism of SC with different structural properties and to help predicting interactions or interpreting toxicological findings in the future. For this purpose, 13 SC were included in the current study. According to their structures, these SC were divided into three subcategories (structures are depicted in Fig. 2). An initial activity screening consisting of incubations with recombinant hCES1b, hCES1c, and hCES2 followed by liquid chromatography-ion trap mass spectrometry (LC–ITMS) analysis was performed to elucidate the involvement of the hCES isoforms in the metabolism of the SC. Furthermore, enzyme kinetics should be modeled if sufficient hydrolysis was observed to compare the substrate specificity of the different human esterases. Finally, the findings should be critically discussed and their impact on the toxicity risk for consumers should be assessed.

## Materials and methods

### Chemicals and reagents

3,5-AB-5F-FUPPYCA (*N*-[(1*S*)-1-(aminocarbonyl)-2-methylpropyl]-1-(5-fluoropentyl)-3-(4-fluorophenyl)-1*H*-pyrazole-5-carboxamide also known as 3,5-5F-AB-FUPPYCA), AB-5F-P7AICA ((*S*)-*N*-(1-amino-3-methyl-1-oxobutan-2-yl)-1-(5-fluoropentyl)-1*H*-pyrrolo[2,3-b]pyridine-3-carboxamide also known as 5F-AB-P7AICA), A-CHMINACA (1-cyclohexylmethyl-*N*-tricyclo[3.3.1.13,7]dec-1-yl-1*H*-indazole-3-carboxamide), DMBA-CHMINACA (2-[1-(cyclohexylmethyl)-1*H*-indazole-3-carboxamido]-3,3-dimethylbutanoic acid), MBA-CHMINACA (2-[1-(cyclohexylmethyl)-1*H*-indazole-3-carboxamido]-3-methylbutanoic acid), MDMB-4F-BINACA (methyl (2*S*)-2-[1-(4-fluorobutyl)-1*H*-indazole-3-carbonyl]amino-3,3-dimethylbutanoate, also known as MDMB-4F-BUTINACA, 4F-MDMB-BUTINACA, or 4F-MDMB-BINACA), MDMB-4en-PINACA (methyl (2*S*)-3,3-dimethyl-2-[(1-pent-4-enylindazole-3-carbonyl)amino]butanoate), MDMB-FUBICA (methyl (2*S*)-2-({1-[(4-fluorophenyl)methyl]-1*H*-indole-3-carbonyl}amino)-3,3-dimethylbutanoate), MDMB-5F-PICA (methyl (2*S*)-2-{[1-(5-fluoropentyl)-1*H*-indole-3-carbonyl]amino}-3,3-dimethylbutanoate also known as 5F-MDMB-PICA), MMB-CHMICA (*N*-[(2*S*)-1-amino-3,3-dimethyl-1-oxobutan-2-yl]-1-(cyclohexylmethyl)-1*H*-indole-3-carboxamide also known as AMB-CHMICA), MMB-4en-PICA (methyl (1-(pent-4-en-1-yl)-1*H*-indole-3-carbonyl)-l-valinate, also known as MMB022), MMB-FUBINACA (*N*-[(2*S*)-1-amino-3,3-dimethyl-1-oxobutan-2-yl]-1-[(4-fluorophenyl)methyl]-1*H*-indazole-3-carboxamide also known as AMB-FUBINACA and FUB-AMB), and MPhP-5F-PICA (methyl (1-(5-fluorpentyl)-1*H*-indole-3-carbonyl)-l-phenylalaninate also known as 5F-MPhP-PICA) were provided by the EU-funded project ADEBAR (IZ25-5793-2016-27). As part of the ADEBAR project, the identity of the compounds was confirmed using different analytical techniques such as gas chromatography–mass spectrometry, (near-)infrared spectroscopy, liquid chromatography–mass spectrometry, Raman spectroscopy, and nuclear magnetic resonance. The content of the samples was determined to be higher than 90% for all samples. AB-PINACA ((*S*)-*N*-(1-amino-3-methyl-1-oxobutan-2-yl)-1-pentyl-1*H*-indazole-3-carboxamide, purity ≥ 98%) was obtained from Cayman Chemicals (Michigan, USA). Thebacon was kindly provided by Prof. Robert Ammon (Homburg, Germany) for previous studies (Meyer et al. 2015). Trimipramine-D3 was obtained from LGC standards (Wesel, Germany). Recombinant hCES1b, hCES1c, and hCES2 (prepared from baculovirus transfected insect cells) as well as pooled human liver microsomes (pHLM, 20 mg microsomal protein/mL, 26 donors) and pooled human liver S9 fraction (pHLS9, 20 mg protein/mL, eight donors) were from Corning (Amsterdam, The Netherlands). After delivery, the enzyme-containing preparations were thawed at 37 °C, aliquoted, snap-frozen in liquid nitrogen, and stored at − 80 °C until use. Potassium dihydrogen phosphate (KH_2_PO_4_), dipotassium hydrogen phosphate (K_2_HPO_4_), and dimethyl sulfoxide (DMSO) were obtained from Merck KGaA (Darmstadt, Germany). All other solvents (analytical grade) were purchased by VWR (Darmstadt, Germany).

Methanolic stock solutions of 3,5-AB-5F-FUPPYCA, AB-5F-P7AICA, A-CHMINACA, DMBA-CHMINACA, MBA-CHMINACA, MDMB-4F-BINACA, MDMB-4en-PINACA, MDMB-FUBICA, MDMB-5F-PICA, MMB-4en-PICA, MPhP-5F-PICA, AB-PINACA (3 mM, each), and MMB-FUBINACA (5 mM), as well as a DMSO stock solutions of MMB-CHMICA (50 mM) and thebacon (100 mM) were used for the initial activity screening experiments. DMSO stock solutions of MMB-CHMICA, MMB-4en-PICA, MPhP-5F-PICA (50 mM, each), MMB-FUBINACA, and thebacon (100 mM, each) were used for the enzyme kinetic studies.

### Initial activity screening with recombinant hCES1b, hCES1c, and hCES2

The initial activity screenings containing 1 of the included 13 SC were carried out as previously described (Meyer et al. 2015). The substrate (final concentration, 100 µM) was incubated for 30 min at 37 °C with hCES1b, hCES1c, hCES2 (final protein concentration, 0.2 µg/µL, each), pHLM, or pHLS9 (final protein concentration, 2 µg/µL, each). The final volume of the incubation mixture was 100 µL, consisting of substrate, enzyme-containing preparation, and phosphate buffer (100 mM, pH 7.4). Negative controls without enzymes were also prepared to monitor non-enzymatic hydrolysis. Thebacon was incubated as positive control to demonstrate suitable incubation conditions. All incubations were done in duplicate. The solvent concentration in the incubations was not higher than 0.2% (DMSO) or 3% (methanol). All reactions were started by adding the enzyme-containing preparation and stopped by adding an equal volume of ice-cold acetonitrile containing 1 µM trimipramine-D3 as internal standard. All samples were centrifuged at 18,407 × *g* for 15 min, 50 µL of the supernatants was transferred to autosampler vials, and 10 µL injected onto the LC–ITMS apparatus for analysis.

The initial activity screening with AB-PINACA was conducted as described by Thomsen et al. with minor modifications (Thomsen et al. 2015). AB-PINACA (final concentration, 10 µM) was incubated for 20 min at 37 °C with hCES1b, hCES1c, hCES2 (final protein concentration, 0.1 µg/µL, each), pHLM, or pHLS9 (final protein concentration, 1 µg/µL, each). The final volume of the incubation mixture was 100 µL, consisting of substrate, enzyme-containing preparation, and phosphate buffer (100 mM, pH 7.4). Negative controls without enzyme were prepared to monitor non-enzymatic hydrolysis. All incubations were done in duplicate. After 20 min, sampling was performed by transferring aliquots of 20 µL to an equal volume of ice-cold acetonitrile, containing 0.5% formic acid and 0.01 µM trimipramine-D3 as internal standard. Afterwards, 110 µL of 0.1% formic acid in water was added. All samples were centrifuged at 2000 × *g*, 5 °C, for 10 min, 50 µL of the supernatant phase were transferred to autosampler vials and 10 µL injected onto the LC–ITMS apparatus. Furthermore, 1 µL was analyzed by liquid chromatography–high-resolution tandem mass spectrometry (LC–HRMS/MS, apparatus details given in the Electronic Supplementary Material).

### Enzyme kinetic studies

Enzyme kinetic studies were conducted to determine kinetic parameters (Michaelis–Menten constant, *K*_m_, and maximum velocity, *V*_max_) using the conditions listed in Table 1. Incubation time and protein concentration were chosen to be within the linear range of metabolite formation and determined in preliminary experiments consisting of incubations with varying incubation time (1–30 min) or protein concentration (0.0125–0.2 µg/µL). Incubation mixtures (final volume, 100 µL) for the enzyme kinetic studies consisted of substrate, enzyme-containing preparation, and phosphate buffer (100 mM, pH 7.4). The DMSO concentration in the incubations ranged from 0 to 4% (2000 µM MMB-4en-PICA with hCES1c). Negative controls without enzyme were prepared to monitor non-enzymatic hydrolysis. All incubations were done in duplicate. The reactions were started by adding the enzyme-containing preparation and stopped by adding an equal volume of ice-cold acetonitrile containing 1 µM trimipramine-D3 as internal standard. All samples were centrifuged at 18,407 × *g* for 15 min, 50 µL of the supernatants was transferred to autosampler vials, and 10 µL injected onto the LC–ITMS apparatus for analysis.

**Table 1:** Enzyme kinetic incubation conditions

Substrate	Enzyme	Incubation time (min)	Protein conc. (µg/µL)	Substrate conc. (µM)
Thebacon	hCES1b	5	0.05	0–3000
	hCES1c	5	0.05	0–2000
	hCES2	5	0.05	0–2000
MMB-CHMICA	hCES1b	15	0.05	0–250
MMB-4en-PICA	hCES1c	30	0.1	0–2000
MMB-FUBINACA	hCES1b	15	0.05	0–500
	hCES1c	10	0.05	0–1000
MPhP-5F-PICA	hCES1b	20	0.05	0–500
	hCES1c	5	0.1	0–250

### ***LC***–***ITMS apparatus***

Apparatus conditions were in line with a previous publication (Meyer et al. 2015). The analytes were separated using a Thermo Fisher Scientific (TF, Dreieich, Germany) Accela ultra-high performance liquid chromatography system consisting of a degasser, a quaternary pump and an autosampler. The LC-system was coupled to a TF LXQ linear ion trap mass spectrometer equipped with a heated electrospray ionization (HESI) II source for analysis. The following LC conditions were used: TF Hypersil GOLD C18 column (100 × 2.1 mm, 1.9 µm particle size) guarded by a TF Hypersil GOLD C18 drop-in guard cartridge and a TF Javelin column filter. Gradient elution was performed with 10 mM aqueous ammonium formate buffer containing 0.1% (v/v) formic acid as mobile phase A and acetonitrile containing 0.1% (v/v) formic acid as mobile phase B. The flow rate was set to 500 µL/min and the gradient was programmed as follows: 0–1 min 98% A, 1–8 min to 30% A, 8–10 min hold 0% A. Re-equilibration of the chromatographic system was performed in a separate run. The following MS conditions were used: ionization mode, positive; sheath gas, nitrogen at flow rate of 34 arbitrary units (AU); auxiliary gas, nitrogen at flow rate of 11 AU; vaporizer temperature, 250 °C; source voltage, 3.00 kV; ion transfer capillary temperature, 300 °C; capillary voltage, 31 V; and tube lens voltage, 80 V. Automatic gain control was set to 15,000 ions for full scan and 5000 ions for MS^n^. For full scan analysis (MS^1^ stage), the maximum injection time was set to 100 ms. Precursor ions were selected from MS^1^ using information-dependent acquisition (IDA) with subsequent collision-induced dissociation-MS^n^ experiments. Hence, MS^1^ was performed in the full scan mode (mass-to-charge ratios, *m/z*, 100–800) and MS^2^ and MS^3^ were performed in IDA mode. The following settings were used for IDA mode: four IDA MS^2^ scan filters were chosen to provide MS^2^ on the four most intense signals from MS^1^, eight MS^3^ scan filters were chosen to record MS^3^ on the two most intensive signals from MS^2^. Furthermore, MS^2^ spectra were collected with higher priority than MS^3^ spectra. Normalized wideband collision energies were set to 35.0% for MS^2^ and 40.0% for MS^3^. Further MS^2^ settings are: minimum signal threshold, 100 counts; isolation width, 1.5 u. Further MS^3^ settings are: minimum signal threshold, 50 counts; isolation width, 2.0 u. Further settings for both stages: activation Q, 0.25; activation time, 30 ms. The dynamic exclusion mode was set as follows: repeat counts, 2; repeat duration, 15 s; exclusion list, 50; exclusion duration, 15 s, and average full scan to full scan cycle time, 4 s.

The TF Xcalibur Qual Browser software version 4.0 was used for data handling. Metabolites formed during the initial activity screening were identified by comparison of their ITMS spectra with reference spectra (Maurer et al. 2019) or by interpretation of the ITMS spectrum fragmentation pattern in comparison to the ITMS spectra of the parent compounds. While full scan mode with IDA was used for analysis of the initial activity screening samples, kinetic study samples were analyzed using full MS^2^ product ion spectra (PIS) of predefined protonated molecules of all target analytes (parent compounds and hydrolysis products) and the internal standard. Monitored masses of the parent compounds and hydrolysis products were *m/z* 342 and 300 for M + H^+^ of thebacon and its metabolite codeine, *m/z* 371 and 357 for M + H^+^ of MMB-CHMICA and its carboxylic acid metabolite, *m/z* 343 and 329 for M + H^+^ of MMB-4en-PICA and its carboxylic acid metabolite, *m/z* 384 and 370 for M + H^+^ of MMB-FUBINACA and its carboxylic acid metabolite, and *m/z* 411 and 397 for M + H^+^ of MPhP-5F-PICA and its carboxylic acid metabolite. The monitored mass of the internal standard trimipramine-D3 was *m/z* 103 (M + H^+^ at *m/z* 298). ChemSketch 2010 12.01 (ACD/Labs, Toronto, Canada) was used for drawing of chemical structures and calculation of masses.

### Data analysis

Data analysis was based on peak areas. Metabolic formation was corrected for non-enzymatic hydrolysis by subtracting any metabolite detected in negative control incubations. For the initial activity screenings, the peak area of the formed metabolite was divided by the parent compound peak area in the negative control and then multiplied by 100 in order to obtain the percentage of substrate, which was hydrolyzed by the respective hCES isoform, pHLM, or pHLS9. Kinetic parameters were calculated using the peak areas of the metabolites, which were divided by the corresponding peak area of the internal standard. Enzyme kinetic constants were determined with nonlinear curve-fitting using GraphPad Prism 5.00 software (San Diego, CA). The Michaelis–Menten equation (see Eq. (1)) is used to calculate K_m_ and V_max_ values for single-enzyme systems (V, velocity; [S], substrate concentration):

1 $$ V = \frac{{V_{\max } \times \left[ S \right]}}{{K_{m} + \left[ S \right]}} $$

In vitro intrinsic clearances (Cl_int_) for the respective metabolic reactions are calculated in accordance with the following equation:

2 $$ {\text{Cl}}_{{\text{int}}} = \frac{{V_{\max } }}{{K_{m} }} $$

## Results

### Initial activity screening experiments

**Fig. 3:**
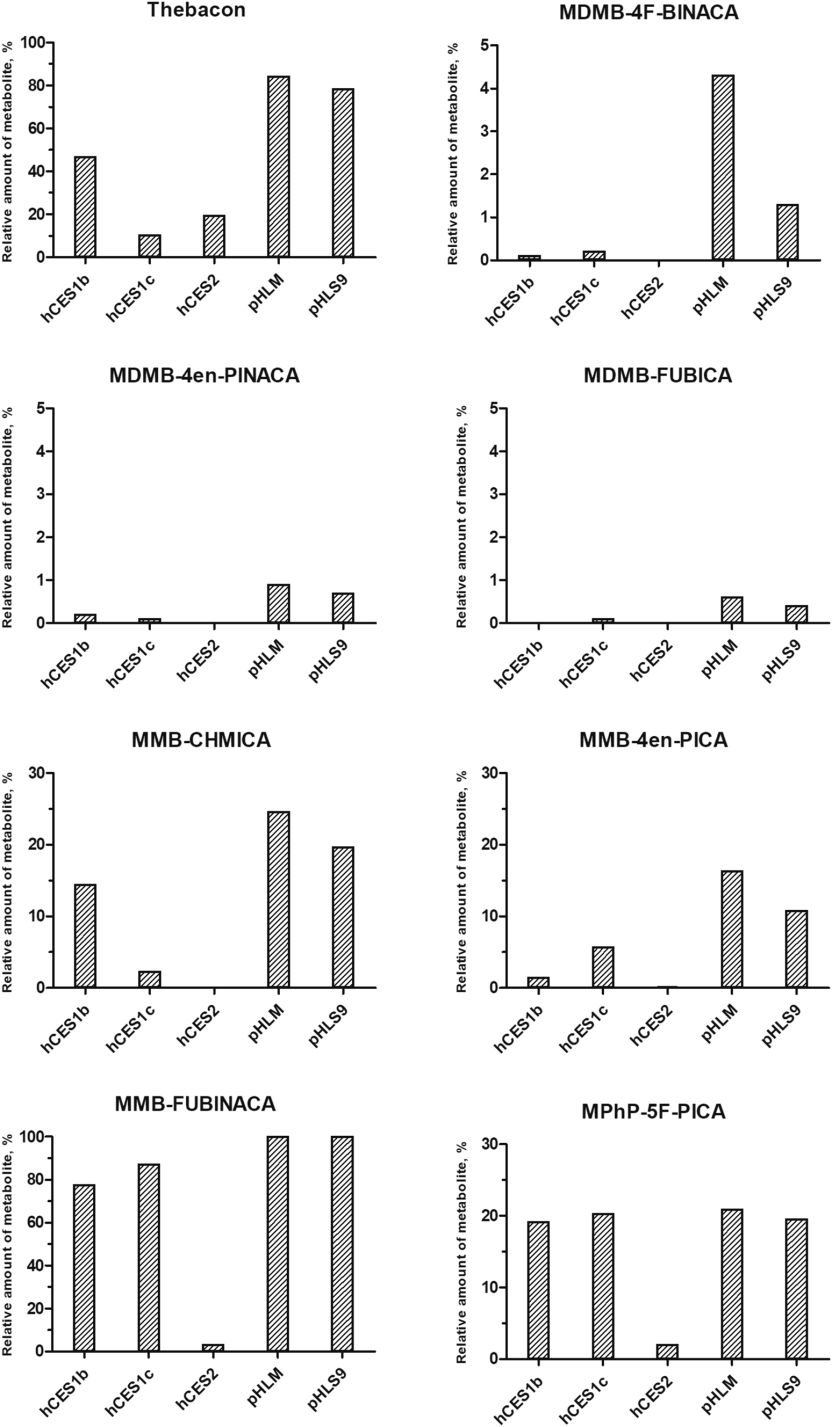
Relative amount formed of metabolites formed after hydrolysis towards remaining parent compounds of all studied drugs of abuse found to be hydrolyzed using different enzyme sources under initial activity screening conditions. Data represents mean of duplicate determination (*n* = 2)

Thebacon, incubated as positive control, was observed to be hydrolyzed by all three hCES isoforms and additionally by pHLM and pHLS9 in vitro. As depicted in Fig. 3, the relative amount of the metabolite codeine formed was 47% for hCES1b, 10% for hCES1c, and 19% for hCES2. In incubations with pHLM and pHLS9, the relative amount of codeine formed was about 80%.

Results of the initial activity screening experiments of the 13 SC are summarized in Table 2. Hydrolysis of the carboxamide linker by hCES, pHLM, or pHLS9 was observed for none of the 13 SC. The carboxamide structure contained in the head group of the SC of subcategory 1 was also found to be not hydrolyzed using the given experimental conditions. The SC of subcategory 2 did not contain a hydrolysable moiety in their head group. However, except for MDMB-5F-PICA, all SC of subcategory 3 with an ester structure in their head group were found to be hydrolyzed by at least one hCES isoform. The ester structures of these seven SC were also found to be hydrolyzed in incubations with pHLM and pHLS9. While hCES1c was involved in the hydrolysis of seven SC and hCES1b of six SC, hCES2 was only involved in the hydrolysis of MMB-4en-PICA, MMB-FUBINACA, and MPhP-5F-PICA. The relative amount of the metabolites formed is depicted in Fig. 3. The extent of hydrolysis was found to differ between MDMB-4F-BINACA, MDMB-4en-PINACA, and MDMB-FUBICA and MMB-CHMICA, MMB-4en-PICA, MMB-FUBINACA, and MPhP-5F-PICA. In case of MDMB-4F-BINACA, MDMB-4en-PINACA, and MDMB-FUBICA, all involved hCES isoforms produced relative amounts of the metabolites below 1%. pHLM and pHLS9 also produced relative amounts of the metabolites of around 1%, except for pHLM and MDMB-4F-BINACA (4.3%). For MMB-CHMICA, hCES1b was found to produce 14% of the carboxylic acid metabolite and hCES1c 2.3%. In contrast, hCES1b only produced 1.5% of the MMB-4en-PICA carboxylic acid metabolite, while hCES1c produced 5.7%, and hCES2 only 0.2%. In case of MPhP-5F-PICA, hCES1b and hCES1c produced similar amounts of the metabolite (19% and 20%, respectively), while hCES2 produced only 2.1%. The amounts of metabolites produced by pHLM and pHLS9 were found to be between 11% (pHLS9 and MMB-4en-PICA) and 25% (pHLM and MMB-CHMICA) for these three SC. Highest relative amounts of metabolite were formed for MMB-FUBINACA, with 78% for hCES1b, 87% for hCES1c, and 3.2% for hCES2. Incubations with pHLM and pHLS9 revealed a complete transformation of MMB-FUBINACA to its carboxylic acid metabolite.

**Table 2:** Initial activity screening results. +, detected; −, not detected; n.a., not applicable

Substrate	Hydrolysis of carboxamide linker			Hydrolysis of carboxamide/ester in head group		
	hCES1b	hCES1c	hCES2	hCES1b	hCES1c	hCES2
3,5-AB-5F-FUPPYCA	−	−	−	−	−	−
AB-5F-P7AICA	−	−	−	−	−	−
A-CHMINACA	−	−	−	n.a	n.a	n.a
DMBA-CHMINACA	−	−	−	n.a	n.a	n.a
MBA-CHMINACA	−	−	−	n.a	n.a	n.a
MDMB-4F-BINACA	−	−	−	**+**	**+**	-
MDMB-4en-PINACA	−	−	−	**+**	**+**	-
MDMB-FUBICA	−	−	−	−	**+**	-
MDMB-5F-PICA	−	−	−	−	−	-
MMB-CHMICA	−	−	−	**+**	**+**	-
MMB-4en-PICA	−	−	−	**+**	**+**	** + **
MMB-FUBINACA	−	−	−	**+**	**+**	** + **
MPhP-5F-PICA	−	−	−	**+**	**+**	** + **

The initial activity screening of AB-PINACA was conducted as described in a previous publication (Thomsen et al. 2015). Using LC–ITMS, no AB-PINACA metabolites were detected. However, the AB-PINACA carboxylic acid metabolite formed after hydrolysis of the carboxamide contained in the head group was detected by LC–HRMS/MS in incubations with hCES1b, pHLM, and pHLS9. The relative amount of metabolite formed accounted for 1.4%, 4.3%, and 2.2%, respectively.

### Enzyme kinetic studies

Calculated enzyme kinetic parameters for the ester hydrolysis of thebacon and four SC are summarized in Table 3. Enzyme kinetic studies were successfully conducted with hCES1b, hCES1c, and hCES2 for thebacon, with hCES1b for MMB-CHMICA, with hCES1c for MMB-4en-PICA, and with hCES1b and hCES1c for MMB-FUBINACA, as well as for MPhP-5F-PICA. Determined K_m_ values for thebacon were 543 µM (hCES2), 655 µM (hCES1b), and 1530 µM (hCES1c), while relative V_max_ values were determined to be 394 AU/min/mg (hCES1b), 482 AU/min/mg (hCES2), and 831 AU/min/mg (hCES1c). Furthermore, hCES-dependent in vitro Cl_int_ are calculated according to Eq. (2) and the relative in vitro Cl_int_ were determined to be between 0.54 AU/min/mg/µM (hCES1c) and 0.89 AU/min/mg/µM (hCES2).

**Table 3:** Calculated enzyme kinetic parameters (± S.D.) for the ester hydrolysis. *K*_m_, Michaelis–Menten constant; *V*_max_, maximum velocity; Cl_int_, in vitro intrinsic clearance

Substrate	Enzyme	*K*_m_ (µM)	*V*_max_ (AU/min/mg)	Cl_int_ (AU/min/mg/µM)
Thebacon	hCES1b	655 ± 210	394 ± 40	0.60
	hCES1c	1530 ± 470	831 ± 140	0.54
	hCES2	543 ± 87	482 ± 31	0.89
MMB-CHMICA	hCES1b	39.3 ± 12	20.8 ± 2.2	0.53
MMB-4en-PICA	hCES1c	203 ± 43	7.57 ± 0.42	0.04
MMB-FUBINACA	hCES1b	40.8 ± 15	70.3 ± 7.6	1.7
	hCES1c	25.7 ± 3.5	30.7 ± 0.96	1.2
MPhP-5F-PICA	hCES1b	88.9 ± 18	182 ± 13	2.0
	hCES1c	23.2 ± 4.1	73.6 ± 3.8	3.2

**Fig. 4:**
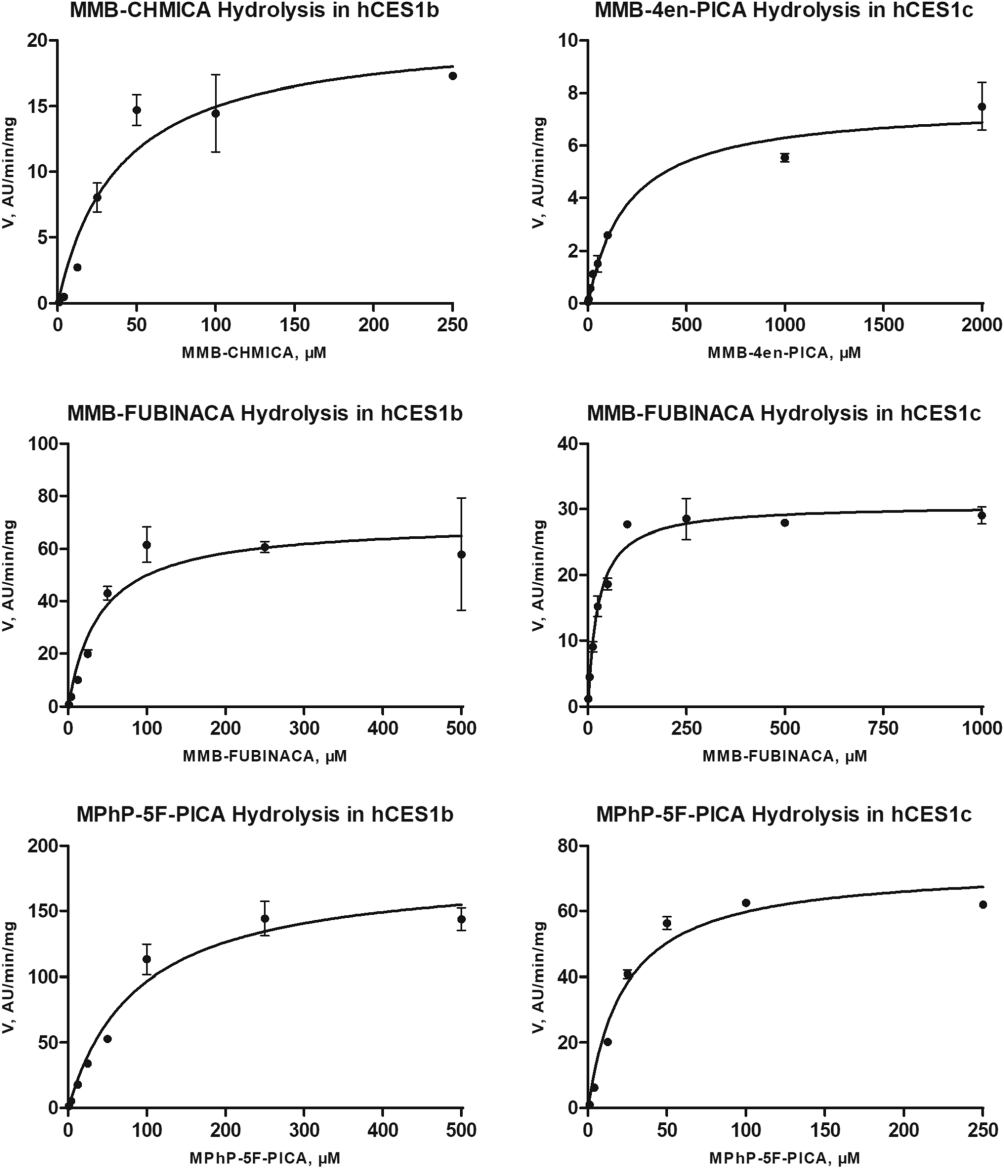
Michaelis–Menten fitted plots for hydrolysis of the studied compounds catalyzed by hCES. Data points represent mean of duplicate determination (*n* = 2). Curves were calculated by nonlinear curve fit according to Eq. (1) (one-site binding model)

As shown in Fig. 4, all SC hydrolysis data fit into Michaelis–Menten kinetics and the resulting K_m_ and V_max_ values, determined according to Eq. (1) for each reaction, are summarized in Table 3. Determined K_m_ values of the hCES1b substrates were determined to be 39.3 µM (MMB-CHMICA), 40.8 µM (MMB-FUBINACA), and 88.9 µM (MPhP-5F-PICA). Determined relative *V*_max_ values ranged from 20.8 AU/min/mg (MMB-CHMICA) to 182 AU/min/mg (MPhP-5F-PICA) resulting in relative in vitro Cl_int_ of 0.53 AU/min/mg/µM (MMB-CHMICA), 1.7 AU/min/mg/µM (MMB-FUBINACA), and 2.0 AU/min/mg/µM (MPhP-5F-PICA). For hCES1c, determined *K*_m_ values were 23.2 µM (MPhP-5F-PICA), 25.7 µM (MMB-FUBINACA), and 203 µM (MMB-4en-PICA). Determined relative *V*_max_ values ranged from 7.57 AU/min/mg (MMB-4en-PICA) to 73.6 AU/min/mg (MPhP-5F-PICA). The lowest relative in vitro Cl_int_ was determined for MMB-4en-PICA with 0.04 AU/min/mg/µM and the highest relative in vitro Cl_int_ for MPhP-5F-PICA with 3.2 AU/min/mg/µM.

## Discussion

### Initial activity screening experiments

Among five hCES subfamilies (Holmes et al. 2010), hCES2 and especially the hCES1 subfamily play an essential role in human drug metabolism (Her and Zhu 2020). Although hCES1 and hCES2 metabolize overlapping substrates, there are differences in terms of the substrate specificity. hCES1 prefers substrates with large acyl moieties and small alcohol parts, whereas hCES2 favors substrates with large alcohol substituents (Imai et al. 2006). An initial activity screening consisting of incubations with recombinant hCES1b, hCES1c, and hCES2 was performed to elucidate the involvement of these hCES isoforms in the metabolism of the SC. Incubations with the human liver cell preparations pHLM and pHLS9 were also included as they contain the natural spectrum of hCES in the human liver.

More than 20 years ago, Chauret et al. described a negative influence of high organic solvent concentrations on recombinant human cytochrome P450 enzyme activities in in vitro incubations (Chauret et al. 1998). In 2008, Williams et al. confirmed the assumption that a similar effect on hCES activities may be possible (Williams et al. 2008). However, solvent concentrations up to 2% were tolerated by hCES1 and hCES2, while hCES1 activities were found to gradually decrease at solvent concentrations above 2%. Therefore, the concentration of the solvents in the current incubations with the recombinant hCES, pHLM, and pHLS9 was chosen as low as possible. However, the limited solubility of the test compounds did not allow incubations free from solvents.

The opiate thebacon was shown to be hydrolyzed by hCES1b, hCES1c, hCES2, pHLM, and pHLS9 in a previous publication and, therefore, incubated as positive control (Meyer et al. 2015). These findings were confirmed by the results of the current study demonstrating suitable incubation conditions.

As shown in Fig. 1, the general structure of SC was described to consist of four subunits, by name core, linker, tail, and head group (Banister and Connor 2018). All studied SC contained a carboxamide linker, which is potentially hydrolyzed by hCES, but different core, tail, and head groups. As the head group of some of the SC additionally contained hydrolysable moieties, this group was used to classify the SC in subcategories as depicted in Fig. 2. The SC of subcategory 1 offered a carboxamide structure in their head group (3,5-AB-5F-FUPPYCA and AB-5F-P7AICA), those of subcategory 2 did not contain a hydrolysable moiety (A-CHMINACA, DMBA-CHMINACA, and MBA-CHMINACA), and those of subcategory 3 exhibited an ester structure derived from a carboxylic acid and methanol (MDMB-4F-BINACA, MDMB-4en-PINACA, MDMB-FUBICA, MDMB-5F-PICA, MMB-CHMICA, MMB-4en-PICA, MMB-FUBINACA, and MPhP-5F-PICA).

The carboxamide structure contained in the head group of the SC of subcategory 1 was not found to be hydrolyzed using the given experimental conditions. As Thomsen et al. reported the hydrolysis of the carboxamide structure in the head group of AB-PINACA by hCES1 and hCES2, their experimental conditions with minor modifications were used to reproduce their findings (Thomsen et al. 2015). Nevertheless, no AB-PINACA metabolites were detected by LC–ITMS. Only LC–HRMS/MS analysis of incubations with hCES1b, pHLM, and pHLS9 enabled the detection of the AB-PINACA carboxylic acid metabolite formed after hydrolysis of the carboxamide contained in the head group. Due to the low relative amounts of metabolite formed, it can be assumed, that the LC–ITMS sensitivity was insufficient. This finding might also explain why the carboxamide in the linker of the 13 SC was not found to be hydrolyzed using the given experimental conditions although Wagmann et al. reported the amide linker hydrolysis of MDMB-4F-BINACA by hCES1c after LC–HRMS/MS analysis (Wagmann et al. 2020). Nevertheless, LC–ITMS was found to be suitable to detect higher hydrolysis rates, crucial for further enzyme kinetic studies. If no hydrolysis was detected by LC–ITMS, it can be assumed that the SC is either not a substrate of the tested hCES isoforms or that the hCES-catalyzed hydrolysis represents only a minor metabolic step. However, it must not be forgotten that these in vitro experiments reflect the conditions in an enclosed environment and that even minor metabolic steps may gain higher importance in vivo. The head group of the SC of subcategory 2 did not contain a hydrolysable moiety and none of the three SC of subcategory 2 was, therefore, used for the subsequent enzyme kinetic studies.

All SC of subcategory 3 with an ester structure, derived from a carboxylic acid and methanol in the head group, except for MDMB-5F-PICA, were found to be hydrolyzed by at least one hCES isoform. However, the extent of hydrolysis was found to be different between MDMB-4F-BINACA, MDMB-4en-PINACA, and MDMB-FUBICA and MMB-CHMICA, MMB-4en-PICA, MMB-FUBINACA, and MPhP-5F-PICA. These findings may be attributed to the structural properties of the SC in subcategory 3, which can be subdivided according to the structures of their head groups. MDMB-4F-BINACA, MDMB-4en-PINACA, MDMB-FUBICA, and MDMB-5F-PICA formed a subgroup with a *tert*-leucine-derived structural motif in their linked group. Another subgroup is formed by MMB-CHMICA, MMB-4en-PICA, and MMB-FUBINACA with a valine-derived structural motif in their linked group. MPhP-5F-PICA has a benzyl group in its secondary moiety. The current results indicate a facilitated hydrolysis for SC with a valine-derived structural motif or a benzyl group. The *tert*-leucine-derived structural motif may be sterically unfavorable in terms of hCES biotransformation. Due to the low relative amounts of metabolites formed in case of SC with a *tert*-leucine-derived structural motif, it cannot be excluded, that MDMB-5F-PICA metabolites were also formed but in amounts below the detection limit. As not all carboxylic acid metabolites of the investigated SC were commercially available, no LC–ITMS detection limits could be determined in the current study.

### Enzyme kinetic studies

Enzyme kinetic studies and calculations of K_m_ and V_max_ values were conducted with those hCES isoforms showing sufficient activity in the initial activity screening. A relative amount of the metabolite formed ≥ 5% under initial activity screening conditions was found to be suitable. This was true for thebacon, MMB-CHMICA, MMB-4en-PICA, MMB-FUBINACA, and MPhP-5F-PICA. The Michaelis–Menten plots depicted in Fig. 4 present the effect of a drug concentration on the velocity of an enzyme-catalyzed reaction and the *K*_m_ represents the drug concentration at which the initial velocity is half maximal (Baranczewski et al. 2006). A low *K*_m_ value indicates a high affinity between enzyme and substrate (Baranczewski et al. 2006). The in vitro Cl_int_ combines *K*_m_ and *V*_max_ values and can be regarded as an indicator for the capacity of an enzyme-catalyzed reaction.

Thebacon was already shown to be hydrolyzed by several hCES and, therefore, used as reference for hCES1b, hCES1c, and hCES2 (Meyer et al. 2015). The current study confirmed the previous findings that hCES1c showed the lowest in vitro Cl_int_, while hCES2 provided the highest in vitro Cl_int_. Nevertheless, the individual kinetic parameters differed from those described in literature. For example, Meyer et al. determined lower *K*_m_ values of 272 µM (hCES1b), 264 µM (hCES1c), and 166 µM (hCES2) for thebacon even though incubation conditions and analyzing methods were identical. A possible explanation for this finding might be the use of hCES loads with different activities. The enzyme activities were determined by the manufacturer using a 4-nitrophenyl acetate assay. In case of hCES1b, the load used by Meyer et al. was described to have a 2.4-fold higher activity than the load used in the current study. For hCES1c, the former activity was 5.3-fold higher and that of hCES2 2.2-fold higher. If the current *K*_m_ values were adjusted by these factors, corrected *K*_m_ values of 273 µM (hCES1b), 289 µM (hCES1c), and 247 µM (hCES2) could be calculated, which were comparable to the former *K*_m_ values. In contrast to the publication by Meyer et al., the metabolites were not quantified using corresponding reference standards. Data analysis was based on the peak area ratios of metabolite and internal standard. As already described before, missing analytical standards for absolute quantification of formed metabolites might be a bottleneck in the assessment of kinetic data (Meyer et al. 2013). Especially metabolites of NPS are often not commercially available. Fortunately, Wagmann et al. were able to demonstrate, that there was no significant difference in the in vitro enzyme contributions to the metabolism of several test drugs calculated via corresponding reference standards or simple peak area ratios (Wagmann et al. 2016). While *K*_m_ values determined via both ways did not differ at all, the use of peak area ratios did only allow the determination of relative *V*_max_ values. However, they were found to be a useful tool for comparison of velocities of different enzymes catalyzing the same reaction (Wagmann et al. 2016). Therefore, the use of peak area ratios should be appropriate for the current study.

Concerning the SC, the relation between substrate structures and enzyme systems will be discussed in the following. As hCES2 only provided low hydrolysis rates of few SC in the initial activity screening, no enzyme kinetic studies with hCES2 and a SC could be performed. However, this finding is in line with literature, as hCES2 was already described to favor substrates with large alcohol substituents (Imai et al. 2006; Meyer et al. 2015). In contrast, hCES1 prefers substrates with large acyl moieties and small alcohol parts and MMB-CHMICA was successfully incubated with hCES1b, MMB-4en-PICA with hCES1c, MMB-FUBINACA and MPhP-5F-PICA with hCES1b and hCES1c, underlining differences in the substrate specificities of both hCES1 isozymes. The affinity of the SC, which are usually smoked by the consumers, to hCES1 is especially noteworthy, because hCES1 was described to be the predominant isoform in the human lung (Di 2019). Among the three hCES1b substrates, MMB-CHMICA and MMB-FUBINACA provided comparably low *K*_m_ values and, therefore, the highest affinities to the enzyme. However, the relative *V*_max_ was higher in case of MMB-FUBINACA, resulting in a higher relative in vitro Cl_int_. For hCES1c, MMB-FUBINACA and MPhP-5F-PICA provided comparably low *K*_m_ values, but the relative *V*_max_ and relative in vitro Cl_int_ were higher in case of MPhP-5F-PICA. The hydrolysis of MMB-FUBINACA and MPhP-5F-PICA were catalyzed by hCES1b and hCES1c. Both SC were shown to have a higher affinity to hCES1c. While the highest relative in vitro Cl_int_ of MPhP-5F-PICA was determined for hCES1c, the relative in vitro Cl_int_ of MMB-FUBINACA in hCES1b incubations was higher than that in hCES1c incubations.

It is obvious that there might be individual pharmacokinetic differences concerning the endogenous ester hydrolysis of the tested compounds. hCES in general are known to be subject to several genetic polymorphism which can lead to interindividual activity differences able to influence the drug metabolism and the clinical outcome of a drug therapy (Merali et al. 2014; Tarkiainen et al. 2012; Zhu et al. 2013). However, an influence on the half-life of drugs of abuse may also be expected. Furthermore, previous studies showed that several natural products such as cannabis or ginsenosides have an impact on hCES1 activity (Qian et al. 2020; Sun et al. 2019). For instance, the three major cannabinoids of *Cannabis sativa*, tetrahydrocannabinol, cannabidiol, and cannabinol, were shown to reduce the hepatic hydrolysis of heroin, which is a substrate of the hCES1 subfamily (Meyer et al. 2015; Qian et al. 2020). Ethanol was shown to inhibit the hydrolysis of the hCES1 drug substrates cocaine and methylphenidate by formation of the transesterification products cocaethylene, a toxic cocaine metabolite, or ethylphenidate, respectively (Redinbo et al. 2003; Zhu et al. 2011). Such an inhibitory effect on the enzyme activity may also be expected for other hCES1 substrates after co-consumption of ethanol. Moreover, hCES are known to be inhibited by various substances such as flavonoids, naturally occurring fatty acids, or organophosphates (Zou et al. 2018). In summary, genetic polymorphisms, drug–food or drug–drug interactions, and other factors are important determinants of the variability in the therapeutic response to drugs hydrolyzed by hCES and may also have an impact on the toxicity risk for consumers of SC. Hence, the role of carboxylesterases in the metabolism of drugs of abuse might also be considered to predict and prevent interactions or interpret toxicological findings.

## Conclusions

The results of this study demonstrate that hCES play an important role in the metabolism of certain SC. The studied SC were divided into three subcategories according to the structural properties of their head groups. After incubation with hCES, metabolite formation could only be detected for SC of subcategory 3, containing an amide linker and an ester bond in the secondary moiety. In general, the metabolite formed was always a product of the ester hydrolysis, mainly catalyzed by hCES1, while the amide linker remained stable under the experimental conditions. Interindividual difference influencing the half-life of the SC caused by hCES1 polymorphisms or drug–drug/drug–food interactions cannot be excluded.

## Supplementary Information

Below is the link to the electronic supplementary material.

Supplementary file1 (PDF 75 KB)
