# Supplementary material for: Going deeper into the toxicokinetics of synthetic cannabinoids: in vitro contribution of human carboxylesterases
Source: Arch Toxicol. 2022 Jul 5;96(10):2755–66. doi: 10.1007/s00204-022-03332-z (PMC9352624; doi:10.1007/s00204-022-03332-z)
Supplement: Supplementary file 1 — Supplementary file1 (PDF 75 KB) [file 204_2022_3332_MOESM1_ESM.pdf]

Electronic supplementary material

Going deeper into the toxicokinetics of synthetic cannabinoids: In vitro contribution of human carboxylesterases

Lea Wagmann, Rebecca G. Stiller, Svenja Fischmann, Folker Westphal, Markus R. Meyer

Liquid chromatography-high-resolution tandem mass spectrometry apparatus

For analysis of the initial activity screening samples of AB-PINACA, apparatus conditions described by Wagmann et al. were adjusted (Wagmann et al. 2020). A TF Dionex UltiMate 3000 RS pump equipped with a degasser, a quaternary pump, and an UltiMate RS autosampler coupled to a TF Q-Exactive Plus system with a HESI II source was used. External calibrations were done prior to analysis as recommended by the manufacturer. The gradient elution was performed on a TF Accucore Phenyl-Hexyl column (100 mm × 2.1 mm, 2.6 µm). Mobile phase A consisted of 2 mM aqueous ammonium formate with 0.1% formic acid (v/v, pH 3) and mobile phase B consisted of 2 mM ammonium formate in acetonitrile:methanol (1:1, v/v) with 1% water (v/v), and 0.1% formic acid (v/v). The gradient was programmed as follows: 0–1 min hold 99% A, 1–10 min to 1% A, 10–11.5 min hold 1%, A and 11.5–13.5 min hold 99% A. The flow rate was set to 500 µL/min for the first 10 min and to 800 µL/min for minutes 10–13.5. The HESI II source conditions were as follows: heater temperature, 320°C; ion-transfer capillary temperature, 320°C; spray voltage, 4.0 kV; ionization mode, positive; sheath gas, 60 arbitrary units (AU); auxiliary gas, 10 AU; sweep gas, 0 AU and S-lens RF level, 50.0. Mass spectrometry experiments were carried out in full-scan mode with subsequent data-dependent MS^2 (ddMS2) with priority set to mass-to-charge ratios (*m/z*) of the parent compound AB-PINACA ($M+H^+$ at *m/z* 331.2128), the two possible metabolites formed after hydrolysis ($M+H^+$ at *m/z* 332.1969 and *m/z* 233.1284), and the internal standard trimipramine-D3 ($M+H^+$ at *m/z* 298.2357). Full-scan data acquisition was performed with the following settings: resolution, 35,000; microscans, 1; automatic gain control (AGC) target, 1e6; maximum injection time (IT), 120 ms and scan range, *m/z* 130–930. For $ddMS^2$ mode an inclusion list was prepared with the previously mentioned *m/z* of AB-PINACA, its expected metabolites, and the internal standard. Furthermore, the following settings were selected: option 'pick others', enabled; dynamic exclusion, 2 s; resolution, 17,500; microscans, 1; isolation window, 1.0 m/z; loop count, 5; AGC target, 2e5; maximum IT, 250 ms; high-collision dissociation cell with stepped normalized collision energy, 17.5, 35.0, 52.5; exclude isotopes, on and spectrum data type, profile. ChemSketch 2010 12.01 was used for drawing of chemical structures and calculation of their exact mass. For data handling the TF Xcalibur Qual Browser software version 4.0 was used.

References

Wagmann L, Manier SK, Bambauer TP, et al. (2020) Toxicokinetics and Analytical Toxicology of Flualprazolam: Metabolic Fate, Isozyme Mapping, Human Plasma Concentration and Main Urinary Excretion Products. J Anal Toxicol 44(6):549-558 doi:10.1093/jat/bkaa019
